# Differential Effects of Autophagy-Related 10 Protein on HCV Replication and Autophagy Flux Are Mediated by Its Cysteine^44^ and Cysteine^135^

**DOI:** 10.3389/fimmu.2018.02176

**Published:** 2018-09-24

**Authors:** Miao-Qing Zhang, Jian-Rui Li, Zong-Gen Peng, Jing-Pu Zhang

**Affiliations:** Key Laboratory of Biotechnology of Antibiotics, National Health Commission (NHC), Institute of Medicinal Biotechnology, Chinese Academy of Medical Sciences and Peking Union Medical College, Beijing, China

**Keywords:** ATG10, cysteine, disulfide bond, autophagy flux, HCV subgenomic replicon, IL28A, lysosomal degradation, structure-function relationship

## Abstract

Autophagy-related 10 (ATG10) is essential for autophagy since it promotes ATG5-ATG12 complex formation. Our previous study found that there are two isoforms of the ATG10 protein, ATG10 (a longer one) and ATG10S, which have identical sequences except an absence of a 36-amino acid fragment (peptide B) in ATG10S, yet exhibit distinct effects on HCV genome replication. Here, we report the existence of two amino acids, cysteine at residue 44 and 135 (Cys^44^ and Cys^135^, respectively), in ATG10 being related to differential effects of ATG10 on HCV replication and autophagy flux. Through a series of ATG10 mutation experiments and protein modeling prediction, we found that Cys^44^ was involved in the dual role of the two isoforms of ATG10 protein on HCV replication and autophagy flux, and that Cys^135^ plays similar roles as Cys^44^, but the disulfide bond of Cys^44^-Cys^135^ was not verified in the ATG10 protein. Further analyses by full HCV virion infection confirmed the roles of -SH of Cys^44^ and Cys^135^ on HCV replication. ATG10 with deleted or mutated Cys^44^ and/or Cys^135^ could activate expression of innate immunity-related genes, including *il28a, irf-3, irf-7*, and promote complete autophagy by driving autophagosomes to interact with lysosomes via IL28A-mediation. Subcellular localization assay and chromatin immunoprecipitation assay showed that ATG10 with the sulfydryl deletion or substitution of Cys^44^ and Cys^135^ could translocate into the nucleus and bind to promoter of IL28A gene; the results indicated that ATG10 with Cys^44^ and/or Cys^135^ absence might act as transcriptional factors to trigger the expression of anti-HCV immunological genes, too. In conclusion, our findings provide important information for understanding the differential roles on HCV replication and autophagy flux between ATG10 and ATG10S, and how the structure-function relationship of ATG10 transformed by a single -SH group loss on Cys^44^ and Cys^135^ in ATG10 protein, which may be a new target against HCV replication.

## Introduction

Autophagy is a major intracellular recycling system in eukaryotes representing the bulk degradation process of cytosolic components by the lysosomal system (1, 2) and plays a crucial role in fundamental biological processes, such as differentiation, development, and cell death (3–6). Autophagy dysfunction is associated with various pathologies, including cancer, infectious diseases, and neurodegenerative disorders (3, 7–9). HCV is a single-stranded positive RNA virus with important roles in chronic liver diseases (10, 11). One interesting phenomena is that autophagy has dual functions on HCV. On the one hand, autophagy plays a positive role in HCV replication by providing autophagic membranes for the replication and assembly of HCV virions (12, 13), or suppressing the host innate immune response to block IFN signaling (14, 15). On the other hand, autophagy can suppress HCV replication via targeting HCV proteins for autophagic degradation (16, 17). To date, how autophagy affects HCV replication remains controversial, and the mechanism of autophagy duality on HCV has not been clearly defined.

In recent years, accumulating evidence has revealed that multiple distinct protein isoforms from a single gene alternative splicing may have different roles in many pathophysiological processes (18–21). Alternative splicing is a mechanism of selecting differential splice sites within pre-mRNA to generate protein diversity that contributes to the specialization of cells and tissues (22, 23). There are quite a few of human autophagy related proteins having alternative spliced isoforms, such as ATG3 (two isoforms), ATG4A-D (each has two isoforms excepting ATG4C), ATG5 (four isoforms), ATG6 (two isoforms), ATG7 (six isoforms), ATG12 (two isoforms) and ATG16L (six isoforms) based on GenBank recording (https://www.ncbi.nlm.nih.gov/nuccore). However, most of the ATG isoforms' functions are unknown besides their predominant isoforms share roles in autophagy. Recently, Niu's group reported that a ATG6 alternative isoform played a dominant negative role on autophagy in leukemogenesis (24). Nevertheless, to the best of our knowledge, whether the other more alternative spliced autophagy-related proteins contributes to the dual function of autophagy and what are their molecular mechanism underlying the dual function have not been reported till now.

Autophagy-related 10 gene (*ATG10*), which encodes an E2-like enzyme (25), interacts with ATG7 to receive an ubiquitin-like protein ATG12, recognizes both ATG12 and ATG5 directly and catalyzes their conjugation reaction (26–28). ATG10 has been shown to play an important role in the invasion and proliferation of cancer cells (8, 29), and bacterial infections (30). At present, the research of the function of ATG10 alone is lacking, and few studies have examined the interdependency between the structure and function of ATG10. Recently, our group provided the first evidence showing that two isoforms of ATG10 protein exist and they were found to have distinct effects on the replication of the HCV subgenome and genome. The shorter isoform (ATG10S) promotes a complete autophagy process, leading to the degradation of HCV-subgenomic replicon and HCV genome. Conversely, the longer isoform (the full-length ATG10) can facilitate HCV-subgenomic replicon amplification by causing impaired autophagy-flux. There is only a 36-amino acid difference between the two isoforms, yet they have opposing effects on HCV subgenome replication (31). Following up on our previous study, we aimed to investigate the mechanism and the specific domains (i.e., the functional sites) that determine the differential roles between the two isoforms. Herein, we attempt to address this question by focusing on the 36-amino acid sequence that is absent in ATG10S by utilizing a series of mutants of ATG10 affecting autophagy flux and HCV replication activity.

## Materials and methods

### Antibodies and reagents

Anti-P62 antibodies for Co-immunoprecipitation (Co-IP) and immunofluorescence (IF) were obtained from MBL (PM045) and Abcam (ab56416), respectively. Anti-LC3B antibodies for Co-IP and IF antibodies were purchased from MBL (PM036). Anti-LAMP2 (sc-18822, sc-20004) was purchased from Santa Cruz Biotechnology for IF and Western blotting. Anti-IL28A (sc-365834) antibody for IF and anti-Lamin B (sc-6216) for Western blotting was purchased from Santa Cruz. Anti-Flag (ab1257) antibody was purchased from Abcam for co-IP and IF. For immunoblot analysis, anti-P62 (PM045) and anti-LC3B (M186-3) were purchased from MBL; anti-ATG10 (AV54274) was obtained from Sigma-Aldrich; antibodies against Hsp90 (ab13495), NS5B (ab122972), CORE (ab2740), NS3 (ab13830), NS4B (ab68632), Flag (ab1257), IL28a (ab38570), IRF3 (ab109255), and IRF7 (ab25950) were obtained from Abcam; and anti-GAPDH and HRP-conjugated goat anti-mouse and goat anti-rabbit IgGs were purchased from ZSGB-BIO Co. (China). For cellular immunofluorescence, the TRITC-labeled goat anti-rabbit IgG, FITC-labeled goat anti-mouse IgG secondary antibodies and mounting medium with DAPI (ZLI-9557) were purchased from ZSGB-BIO (China). For IP, rabbit IgG (A7016) and mouse IgG (A7028) were purchased from Beyotime Biotechnology; protein A/G plus agarose (sc-2003) was obtained from Santa Cruz Biotechnology. Lipofectamine 2000 Reagent was purchased from Invitrogen. Protein extraction reagent RIPA lysis buffer (C1053), non-denaturing lysis buffer (C1050) and protease inhibitor (cocktail, 50x, P1265-1) were purchased from Applygen Technologies, Inc. (China).

### Plasmids

The fragment deletion mutants of ATG10^Δ1^, ATG10^Δ1−2^, ATG10^Δ2−3^, ATG10^Δ3−4^, ATG10^Δ4−5^, ATG10^Δ5−6^, and ATG10^Δ6^ in pIRES2-EGFP were synthesized by Sangon Biotech Co. (Shanghai, China). The amino acid deletion within M^43^-C^44^-K^45^-I^46^-H^47^-F^48^ (unit-2) was performed sequentially in ATG10 via deleting each triplet code and it was constructed in pIRES2-EGFP vector, which formed six mutants with a single amino acid deletion: ATG10^ΔM43^, ATG10^ΔC44^, ATG10^ΔK45^, ATG10^ΔI46^, ATG10^ΔH47^, and ATG10^ΔF48^. The plasmids of pIE-ATG10/ATG10S and of the ATG10 mutants with methionine at 43 site (Met^43^)- or cys^44^-deletion and with Cys^44^ and Cys^135^ substitution (C44A, C44S, C135S, and C44S/C135S) mutations were constructed into pIRES2-EGFP in our laboratory. The mutations of ATG10 in Cys^44^ and Cys^135^ were performed by using overlap-PCR. All of the ATG10 mutants were Flag-tagged at their N-terminals and identified by sequencing.

A series of N- or C-terminally truncated ATG10 mutants across the whole ATG10 length were constructed based on six exons of *atg10*; the six exons were deleted from the 5′- terminal or 3′-terminal sequentially and inserted into pIRES2-EGFP vector, forming six truncated mutants: ATG10^2−6^, ATG10^3−6^, ATG10^4−6^, ATG10^1−3^, ATG10^1−4^, and ATG10^1−5^.

### Cell lines and HCV subreplicon

HepG2 cells were purchased from National Infrastructure of Cell Line Resource. Cells were cultured in MEM (Gibco) supplemented with 10% fetal bovine serum at 37°C in a 5% CO_2_ incubator. The HCV subgenomic replicon sequence was cloned from HCV 1b genomic sequence and the subreplicon was constructed in two plasmids p5BR and prGC3N described in a previous work (32, 33). p5BR expresses HCV RNA-dependent RNA polymerase NS5B, and prGC3N expresses complementary sequences of HCV 5′UTR-core and 3′UTR as a HCV RNA subgenomic template.

### Overexpression of ATG10 mutants in HCV subgenomic replicon cells

The HCV subgenomic replicon cells were constructed using p5BR and prGC3N co-transfected into HepG2 cells, and after 6 h, each pIE-ATG10 mutant plasmid was transfected into the HCV subgenomic replicon cells, and the cells were collected for subsequent experiment after 40–48 h. Transient transfection was performed using Lipofectamine 2000 reagent according to the manufacturer's instructions.

For overexpressing N- or C-terminally truncated ATG10 mutants, based on six exons of *atg10*, 5′-capped mRNAs of ATG10 truncated mutants were synthesized *in vitro* using a capped mRNA kit (Ambion, AM1348) with the truncated-ATG10 vectors (pGEM-T-ATG10^2−6^, -ATG10^3−6^, -ATG10^4−6^, -ATG10^1−3^, -ATG10^1−4^, and -ATG10^1−5^) as the templates. Each 100 ng 5′-capped mRNA was transfected into the HCV subgenomic replicon cells. Then, the cells were collected after a 40–48 h of culture for subsequent experiments. The overexpression level of *atg10, atg10s, atg10*^1−3^, *atg10*^1−4^, *atg10*^1−5^, *atg10*^2−6^, *atg10*^3−6^, or *atg10*^4−6^ genes were analyzed by RT-PCR, the primer sequences for RT-PCR (5′-3′) are as follows: *atg10* or *atg10S* (F-ATGGAAGAAGATGAGTTCATTGG, R-TTAAGGGACATTTCGTTCATCCTGAG); *atg10*^2−6^ (F-ATGGACTGTTCTGATGGCTACATGTG, *atg10-*R); *atg10*^3−6^ (F-ATGGAGGCTTTCGAGCTACCCTTGGA, *atg10-*R); *atg10*^4−6^ (F-ATGGATGGGAGACCTTTAACTCTGAA, *atg10-*R); *atg10*^1−5^ (*atg10-*F, R-TTACTTATTGATTTTCTGAGAATTCT); *atg10*^1−4^ (*ATG10-*F, R-TTACTGTTGCGTAATAGTGTCCCATG); *atg10*^1−3^ (*atg10-*F, R-TTAATCTAAAAAGCTTGCCCTAAAGT); β*-actin* (F-AGGGAAATCGTGGGTGACATCAAA, R-ACTCATCGTACTCCTGCTTGCTGA).

### Influence of rapamycin on the protein level of immune factors

HepG2 cells was transfected with the plasmid mixture of prGC3N and p5BR and cultured for 6 h, then were secondly transfected with the plasmid of ATG10, ATG10S, and ATG10 mutants respectively and cultured for 24 h. Then the cells were exposed to RAPA (50 nM) for another 24 h. The cells were collected and examined protein levels of the immune factors (IL28A, IRF3, and IRF7) and autophagy flux-related proteins (LC3B and P62) using western blot analysis.

### Immunoprecipitation and immunofluorescence

For IP, the HCV subgenomic replicon cells transiently transfected with the plasmids of ATG10 mutants were lysed with non-denaturing lysis buffer and protease inhibitor cocktails. The lysate was precleared with protein A/G plus agarose beads at 4°C for 30 min and incubated with anti-P62, anti-LC3B, anti-LAMP2, or anti-Flag antibody at 4°C overnight, and then with protein A/G plus agarose at 4°C for 2 h. The agarose was washed 3 times with RIPA lysis buffer or PBS and eluted with SDS loading buffer by boiling for 6 min. Boiled samples were subjected to immunoblot analysis.

For IF, the HCV subgenomic replicon cells were seeded on coverslips and transfected with the plasmids of ATG10 mutants for 40–48 h, and then cells fixed with 1% paraformaldehyde for 15 min at room temperature. After washing 3 times with PBS, cells were permeabilized with 0.5% Triton X-100 for 10 min, then probed with anti-P62, anti-LC3B, anti-LAMP2, anti-Flag, or anti-IL28A antibodies at 4°C overnight. After 3 washes with PBS, the cells were incubated with secondary antibodies labeled with TRITC or FITC (1:100 dilutions) for 1 h. Next, the cells were counterstained with DAPI dye in mounting medium and observed under a DeltaVision Imaging System (GE Healthcare). In HCV subgenomic replicon cells, FITC-anti-LC3B and TRITC-anti-P62 antibodies were used for the conjunction of p62-cargos with autophagosomes. FITC-anti-LC3B and TRITC-anti-LAMP2 antibodies were used for the conjunction of autophagosomes with lysosomes.

### HCV virion infection

HCV virion infection was performed as previously described (31). Briefly, Huh7.5 cells were separately transfected with a designed concentration of ATG10, ATG10S, ATG10^ΔM43^, ATG10^ΔC44^, ATG10^C44S^, ATG10^C44A^, ATG10^C135S^ and ATG10^C44S/C135S^ using Lipofectamine 2000 (Invitrogen). After 6 h, the culture supernatants were replaced with fresh complete culture media, and then infected with HCV virions (HCV 2a, J6/JFH/JC, 45 IU/cell) for 72 h. Total proteins and RNAs were extracted and detected with Western blotting and qPCR, respectively.

### Real-time qPCR

Total RNA was isolated with TRIzol reagent and reverse-transcribed with AMV reverse transcriptase (Promega). The generated cDNA1st strand was used for qPCR, and amplification of *il-28a, irf-3, irf3*, and β*-actin* was performed by Roche 480. Results were analyzed using the comparative ΔΔCt method. The primer sequences for qPCR (5′-3′) are as follows: *irf-3* (F-TCTGCCCTCAACCGCAAAGAAG, R-TACTGCCTCCACCATTGGTGTC); *irf-7* (F-CCACGCTATACCATCTACCTGG, R-GCTGCTATCCAGGGAAGACACA); *il-28a* (F-TCGCTTCTGCTGAAGGACTGC, R-CCTCCAGAACCTTCAGCGTCAG); *core* (F-CAACCTCGTGGAAGGCGACAAC, R-GGACAGCAGAGCCAAGAGGAAGATAG); β*-actin* (F-CACCATTGGCAATGAGCGGTTC, R-AGGTCTTTGCGGATGTCCACGT).

### IL28A downregulation in the HCV subgenomic replicon cells

The IL-28A morpholino oligomer sequences (Gene Tools, LLC) are 5′-TTCATTCCT GATCTCTGGTCTTTGT-3′ (MO1) and 5′-AAACACTCTGAGGCTGTCACCCAGG-3′ (MO2). These oligomers complementary differentially to *IL28A* mRNA 5′UTR sequence around (MO1 covers the start codon ATG). IL28A-knockdown was carried out by morpholino transfection at concentrations of 100 pM for each well in the HCV subgenomic replicon cells. Then, the cells were cultured for 40–48 h and collected for the subsequent tests.

### Nuclei–cytoplasm fractionation experiment

HCV subgenomic replicon cells were separately transfected with a designed concentration of Flag-tagged ATG10, ATG10S, ATG10^ΔM43^, ATG10^ΔC44^, ATG10^C44S^, ATG10^C44A^, ATG10^C135S^ and ATG10^C44S/C135S^, and the cells were collected for subsequent experiment after 40–48 h. Nuclei–cytoplasm fractionation was conducted using the NE-PER Nuclear and Cytoplasmic Extraction Reagents kit (Thermo Fisher Scientific) according to the manufacturer's protocol. Western blotting was performed to detected ATG10, ATG10S, and ATG10 mutants in the seperated nuclei and cytoplasm separately using anti-Flag antibody. Lamin B and Hsp90 served as the nuclear marker and cytoplasmic marker, respectively.

### Chromatin immunoprecipitation (ChIP) assay

ChIP assay was performed using the ChIP Assay kit (Beyotime Institute Biotechnology, China) according to the manufacturer's protocol. Briefly, to HCV subgenomic replicon cells transfected separately with Flag-tagged ATG10, ATG10S, ATG10^ΔM43^, ATG10^ΔC44^, ATG10^C44S^, ATG10^C44A^, ATG10^C135S^, and ATG10^C44S/C135S^ culture, 1% formaldehyde was added. The culture was incubated at 37 °C for 10 min to allow cross-linking of proteins and DNA. Following three times of wash with cold PBS supplemented with 1 mM PMSF, the cells were resuspended using a buffer containing 1% SDS and 1 mM PMSF, and lysed by sonication. After centrifugation, the supernatant was collected and the chromatin in the supernatant was immunoprecipitated with anti-Flag. Flag-immunoprecipitated DNA was amplified with PCR using specific primers to analyze the ATG10 mutants binding site of the putative il28a promoter. The il28a promoter primers were described as follows: primer 5′-CGTGGTGGTGCATGCCTATA-3′ and reverse primer 5′-TAACTGCAACCTCCACCTCC-3′.

### Protein homology modeling

Steric conformations of ATG10, ATG10S, and the mutated ATG10 proteins were modeled using the SWISS MODEL software online (http://swissmodel.expasy.org/) and the template 4gsk.1.c (hetero-2-2-mer, and by a method of X-RAY DIFFRACTION 2.90 Å). The SWISS-MODEL template library (SMTL version 2018-01-31, PDB release 2018-01-26) was searched with BLAST (33) and HHBlits (34) for evolutionary-related structures matching the target sequence. Template search was based on previously published literature (4, 35, 36).

### Statistical analysis

Statistical analysis were performed using GraphPad Prism 7 software. Data shown are mean ± SD, the means and standard deviations in histograms are derived from three independent experiments. The one-way analysis of variance (ANOVA) tests was used for all data sets, and *P*-values < 0.05 were considered as signifcant.

## Results

### Identification of a functional domain in peptide-B of ATG10 protein

**Figure 1:**
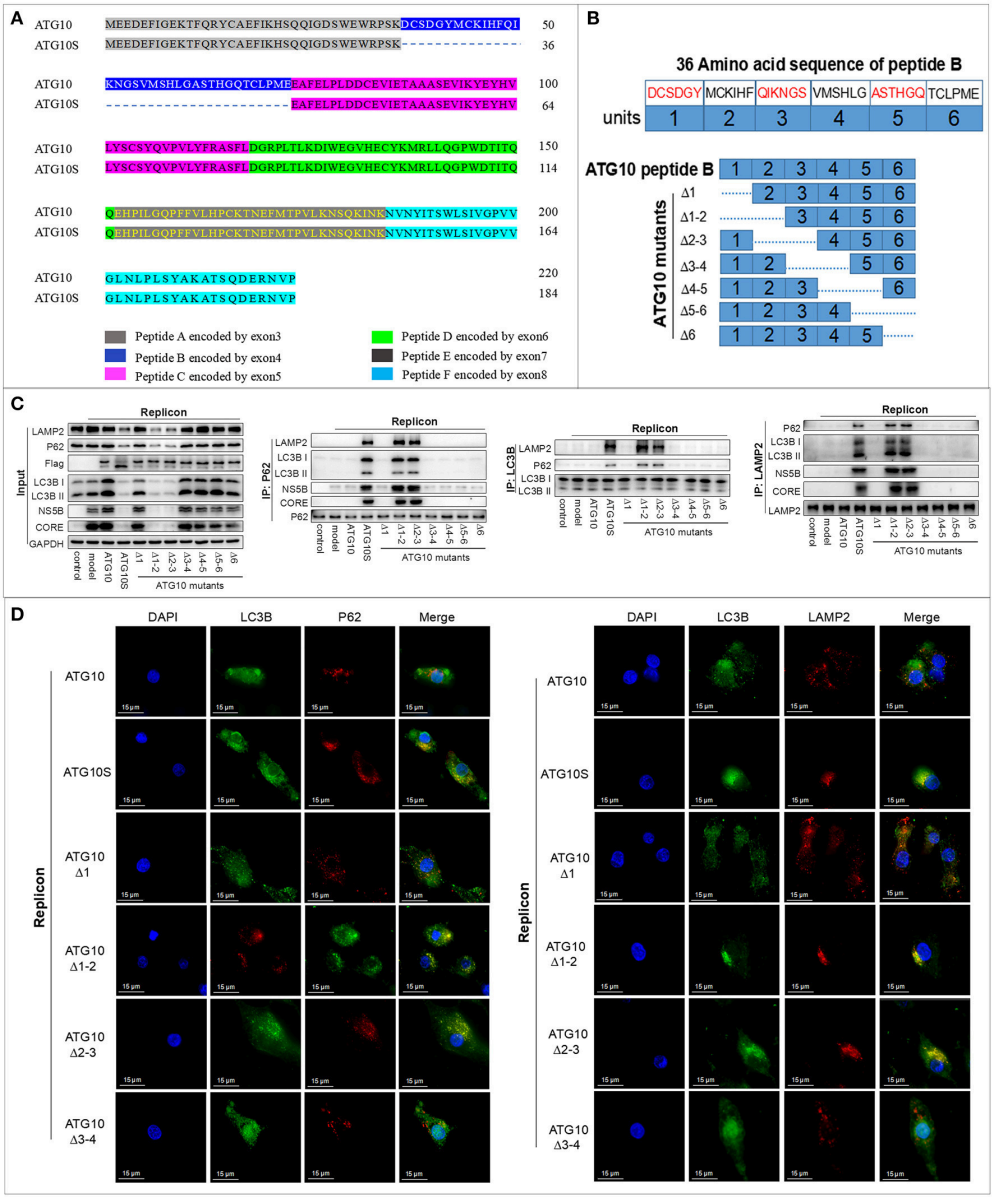
The functional domain was identified in peptide-B of the ATG10 protein. **(A)** The amino acid sequence alignment of ATG10 and ATG10S encoded by the *atg10* transcript variant 3 sequence in human chromosome 5. **(B)** The diagram of seven ATG10 deletion-mutants in peptide-B. The dotted lines represent the deletion parts. **(C)** Co-immunoprecipitation analysis of interactions among LC3B, P62, and LAMP2 in the HCV-subgenomic replicon cells transfected by the seven ATG10 mutants, respectively. **(D)** Subcellular localization of P62-LC3B and LC3B-LAMP2 in ATG10, ATG10S, ATG10^Δ1^, ATG10^Δ1−2^, ATG10^Δ2−3^, or ATG10^Δ3−4^ overexpressed HCV subreplicon cells by immunofluorescence. Scale bars, 15 μm.

In our previous study, we found that ATG10 and ATG10S presented distinct effects on HCV genome replication and have nearly 100% identical sequences except for the absence of a 36-amino acid fragment in the ATG10S isoform (Figure 1A). The 36-amino acid polypeptide is encoded by exon-4 of the *atg10* transcript variant 3 sequence in human chromosome 5 and its absence was shown to cause a decrease of HCV subgenomes by autophagy (31). Thus, we speculated that a functional site exists within the 36-amino acid sequence. We divided the 36 amino acids into six equal fragments. Deletion of the DNA sequence coding the six amino-acid fragments from the N terminal was sequentially performed to generate seven ATG10 deletion mutants (Figure 1B).

To test the influence of the seven deletions on autophagy flux, HCV subgenome replication cells were transiently transfected with the deleted ATG10 expression constructs. Co-IP assay revealed that under ATG10^Δ1−2^ or ATG10^Δ2−3^ overexpression, P62 significantly interacted with both LC3B and LAMP2, one of the lysosomal membrane proteins. Additionally, LC3B interacted with LAMP2 and P62 proteins, which was similar to ATG10S overexpression (Figure 1C). Importantly, HCV CORE and NS5B proteins combined with P62 and LAMP2 proteins, meaning that the HCV proteins were enwrapped in autophagosomes. Meanwhile HCV CORE, NS5B, P62, and LC3B were notably reduced in the input results, which indicated that the HCV proteins were degraded via autolysosomes, similar to the ATG10S group. The other ATG10 deletions exhibited similar results as the ATG10 overexpression and were opposite to ATG10S overexpression. Further, in the subcellular co-localization experiments, both co-localized particles of P62-LC3B and LC3B-LAMP2 were aggregated around the nuclei in both the ATG10^Δ1−2^- and ATG10^Δ2−3^-expressing cells, while no co-localization was evident in the ATG10^Δ1^- and ATG10^Δ3−4^-expressing cells (Figure 1D; Figure S1D). These results suggested that the virus proteins were degraded in autolysosomes, and ATG10^Δ1−2^ and ATG10^Δ2−3^ played similar roles as ATG10S in promoting complete autophagy, resulting in the lysosomal degradation of HCV subgenomic replicon.

As shown on Figure 1B, ATG10^Δ1−2^ and ATG10^Δ2−3^ shared a common deletion fragment containing the amino acid sequence of M^43^-C^44^-K^45^-I^46^-H^47^-F^48^ known as unit-2 here, and presented similar biological functions. We posit that the effect of the ATG10 mutant with unit-2 deletion on autophagy flux and on HCV subreplicon was similar to ATG10S. Furthermore, *atg10s* sequence lacks the peptide-B encoded by the exon-4 of *atg10*, which covers the unit-2 sequence M^43^-C^44^-K^45^-I^46^-H^47^-F^48^. Therefore, we infer that the functional amino acid fragment is M^43^-C^44^-K^45^-I^46^-H^47^-F^48^ in peptide-B.

### Cysteine at 44 site is a critical amino-acid residue within the unit-2 of ATG10 for autophagy flux and HCV subreplicon degradation

**Figure 2:**
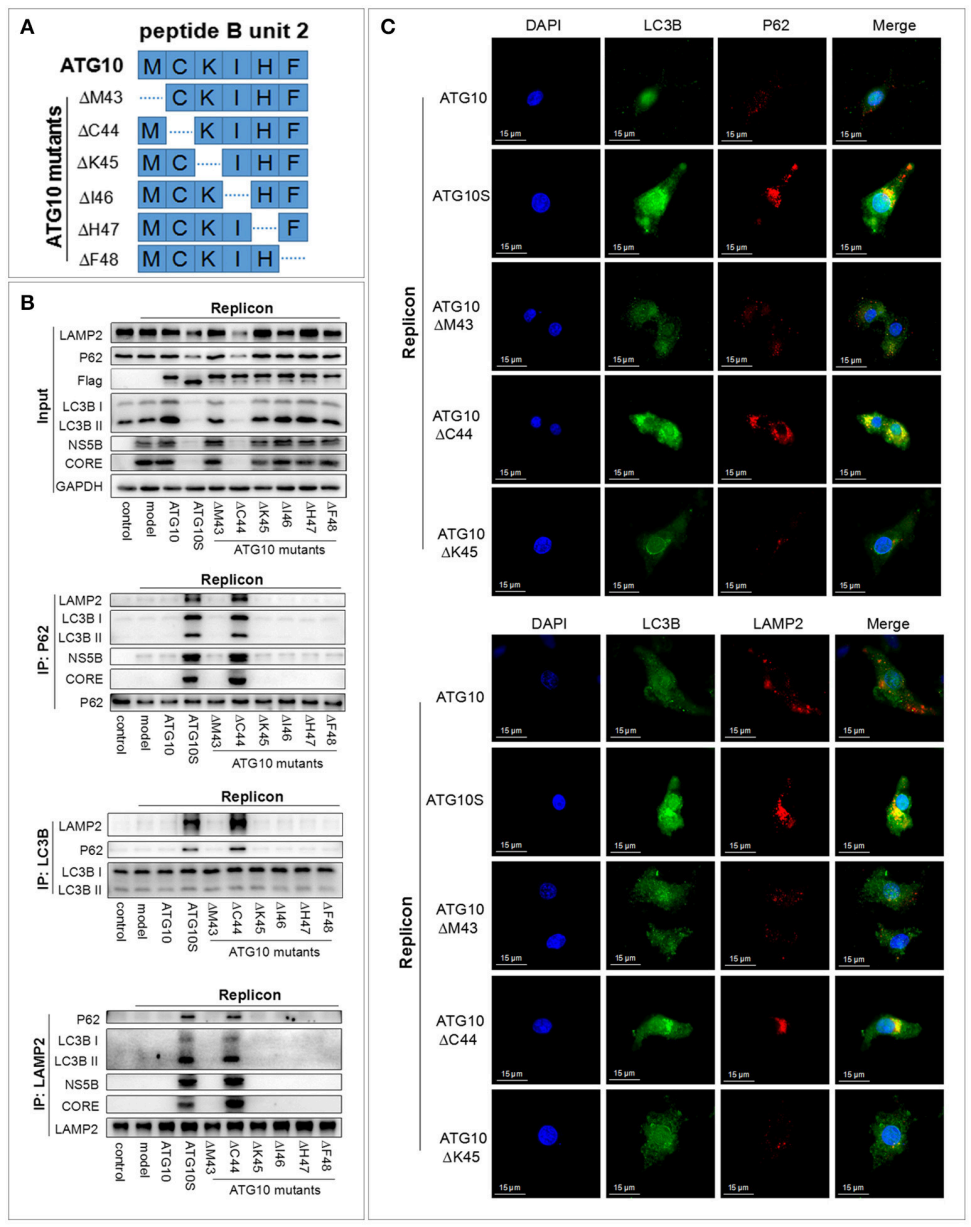
Cysteine at 44 site of the ATG10 protein is a crucial amino acid residue for ATG10 protein. **(A)** The construction diagram of six ATG10 mutants in M^43^-C^44^-K^45^-I^46^-H^47^-F^48^. The dotted lines represent deletion sites. **(B)** Co-immunoprecipitation with anti-LC3B, anti-P62, and anti-LAMP2 in HCV-subgenomic replicon cells, and analysis of the effect on expression levels of HCV subreplicon proteins and autophagy proteins by Western blotting. **(C)** Subcellular co-localization of P62-LC3B and LC3B-LAMP2 under ATG10, ATG10S, ATG10^ΔM43^, ATG10^ΔC44^, or ATG10^ΔK45^ overexpression in HCV subgenomic replicon cells by immunofluorescence. Scale bars, 15 μm.

Since the functional fragment unit-2 of the six amino acid fragments was found, we investigated whether any single amino acid played the critical role. Amino acid deletion within M^43^-C^44^-K^45^-I^46^-H^47^-F^48^ was performed sequentially in ATG10 via deleting each triplet code in six expressing vectors, respectively (Figure 2A). The results of co-IP and IF showed that only ATG10^ΔC44^ of the six vectors could induce the combination of P62 with both LC3B and LAMP2, and LC3B with LAMP2 and P62 (Figures 2B,C; Figure S2C). These results suggested that the ATG10 variant with cysteine^44^ deletion promoted the transportation of autophagosomes to lysosomes and the formation of autophagolysosomes. Simultaneously, the levels of autophagy proteins (P62, LC3B, and LAMP2) and HCV subgenomic replicon proteins (NS5B and CORE) were decreased significantly in the HCV subreplicon plus ATG10^ΔC44^-expressing cells compared to the other five mutants (Figure 2B). Furthermore, co-IP experiments showed that NS5B and CORE proteins were co-precipitated with P62 and LAMP2 in HCV subgenomic replicon cells with only the ATG10^ΔC44^ expressive vector (Figure 2B). These results verified that the virus proteins were transferred into autolysosomes by ATG10^ΔC44^ expression.

The data above indicated that ATG10^ΔC44^ promoted the degradation of HCV sugenomic replicon by inducing a complete autophagic flux. The similar results were obtained in ATG10S-overexpressing cells. Therefore, we inferred that the critical amino acid residue within M^43^-C^44^-K^45^-I^46^-H^47^-F^48^ was cysteine at 44 site (Cys^44^) of the ATG10 protein. This cysteine deletion ensures that the ATG10 protein mimics ATG10S action on HCV replication and on autophagy flux.

### Hydrosulphonyl of Cys^44^ is a critical group determining the differential roles of ATG10 and ATG10S

**Figure 3:**
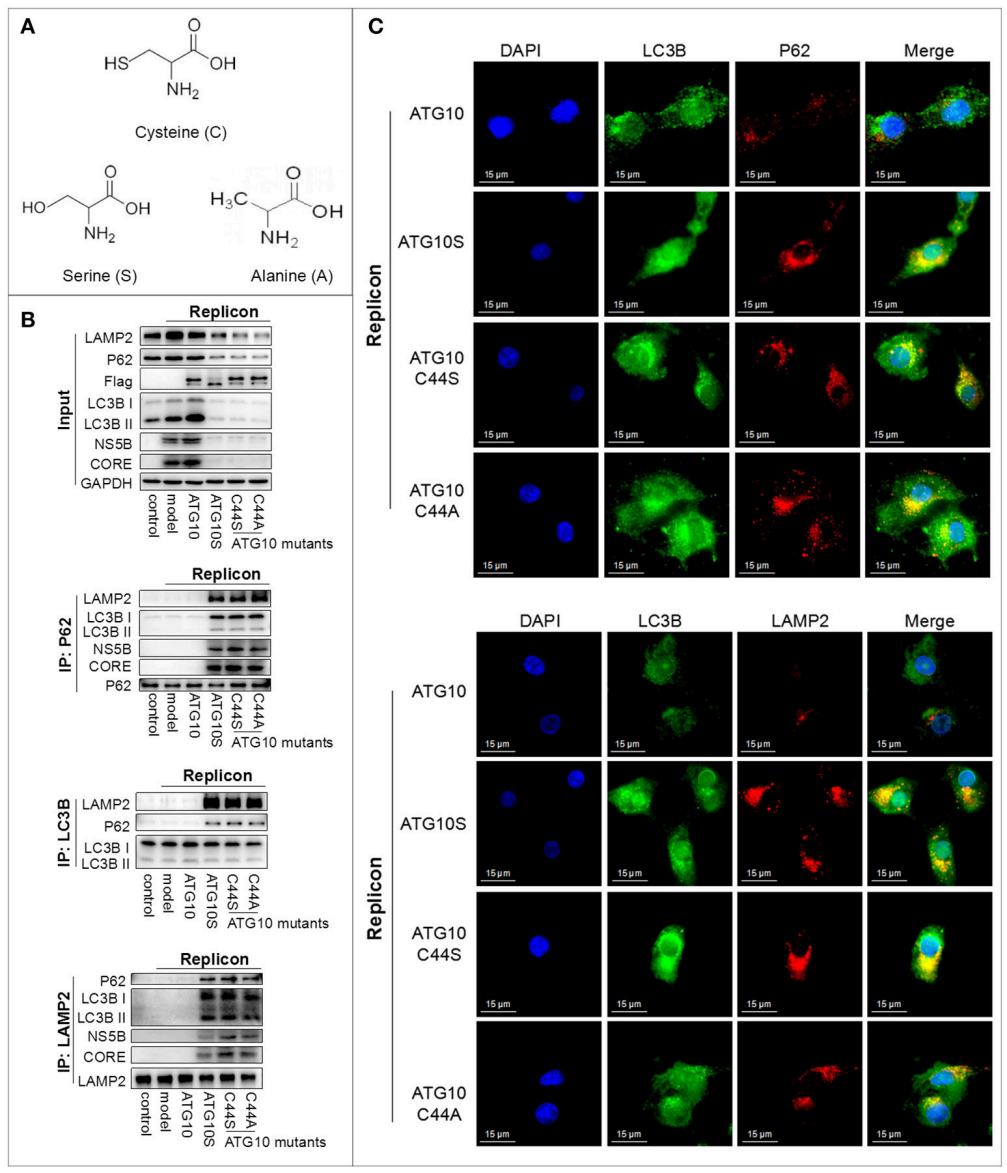
The hydrosulphonyl of Cys^44^ is a critical group determining the differential roles of ATG10 and ATG10S. **(A)** The structure of cysteine, serine, and alanine amino acids. ATG10^C44S^ and ATG10^C44A^ mutants in which cysteine was replaced with serine or alanine, the –SH group converted into –OH or –H, respectively. **(B)** Co-immunoprecipitation showed the protein levels and interaction among LC3B, P62, LAMP2, HCV CORE, and NS5B proteins in HCV-subgenomic replicon cells induced by ATG10S, ATG10, and ATG10^C44S^ and ATG10^C44A^ mutants. **(C)** Subcellular co-localization of P62-LC3B and LC3B-LAMP2 under ATG10, ATG10S, ATG10^C44S^, and ATG10^C44A^ overexpression in the HCV subgenomic replicon cells by immunofluorescence. Scale bars, 15 μm.

The specific chemical group in the side chain of cysteine is hydrosulphonyl (–SH), which can form the disulfide bond to maintain the structure and function of proteins. In order to identify the –SH key roles of Cys^44^ in ATG10 to suppress HCV genome replication, we constructed ATG10^C44S^ and ATG10^C44A^ mutants in which the cysteine was replaced with serine or alanine, and the –SH group was converted into –OH or –H, respectively (Figure 3A). To test the effect of ATG10^C44S^ and ATG10^C44A^ on autophagy flux, we examined the interaction and the subcellular localization among autophagy proteins (P62, LC3B, and LAMP2) by co-IP and IF (Figures 3B,C) in ATG10^C44S^- or ATG10^C44A^-overexpressing HCV-subreplicon cells. Similar to the ATG10S group, the protein levels of P62, LC3B, LAMP2, HCV CORE and NS5B significantly decreased, and combinations of P62 with HCV CORE, NS5B, LC3B and LAMP2, and of LAMP2 with LC3B, P62, HCV CORE and NS5B were notably increased compared with the HCV-model and HCV-model plus ATG10 groups. These results indicated that the ATG10^C44S^ and ATG10^C44A^ mutants could trigger the complete autophagy process by promoting the formation of autophagosomes and their maturation into autolysosomes and HCV proteins were transferred into autolysosomes for degradation. The subcellular co-localization experiments confirmed the complex consisted of LC3B, P62, and LAMP2, which co-localized and accumulated around the nuclei, and was greatly induced by the overexpression of ATG10^C44S^ and ATG10^C44A^ mutants other than by native ATG10 (Figure 3C; Figure S3C). Taken together, these data suggested that ATG10^C44S^ and ATG10^C44A^ could induce complete autophagy flux, resulting in the degradation of the HCV subgenomic replicon.

Since the action of ATG10^C44S^ and ATG10^C44A^ were consistent with ATG10S, we hypothesized that the –SH of Cys^44^ plays a crucial role on the differential roles of ATG10 and ATG10S. Although the three amino acids, cysteine, serine, and alanine, have the same carbon chain and only one atom difference on their side group: -OH with the similar polarity property to –SH or –H of nonpolar alanine, both substitution of Cys^44^ changed the ATG10 function to the ATG10S function. These results suggested that the specific role of Cys^44^ might be dependent on its hydrosulphonyl group, which contributes to the formation of disulfide bonds.

### Cys^44^ and Cys^135^ potentially form a disulfide bond and their functional role in transformation of ATG10 protein in autophagy flux and suppression of HCV genome replication

**Figure 4:**
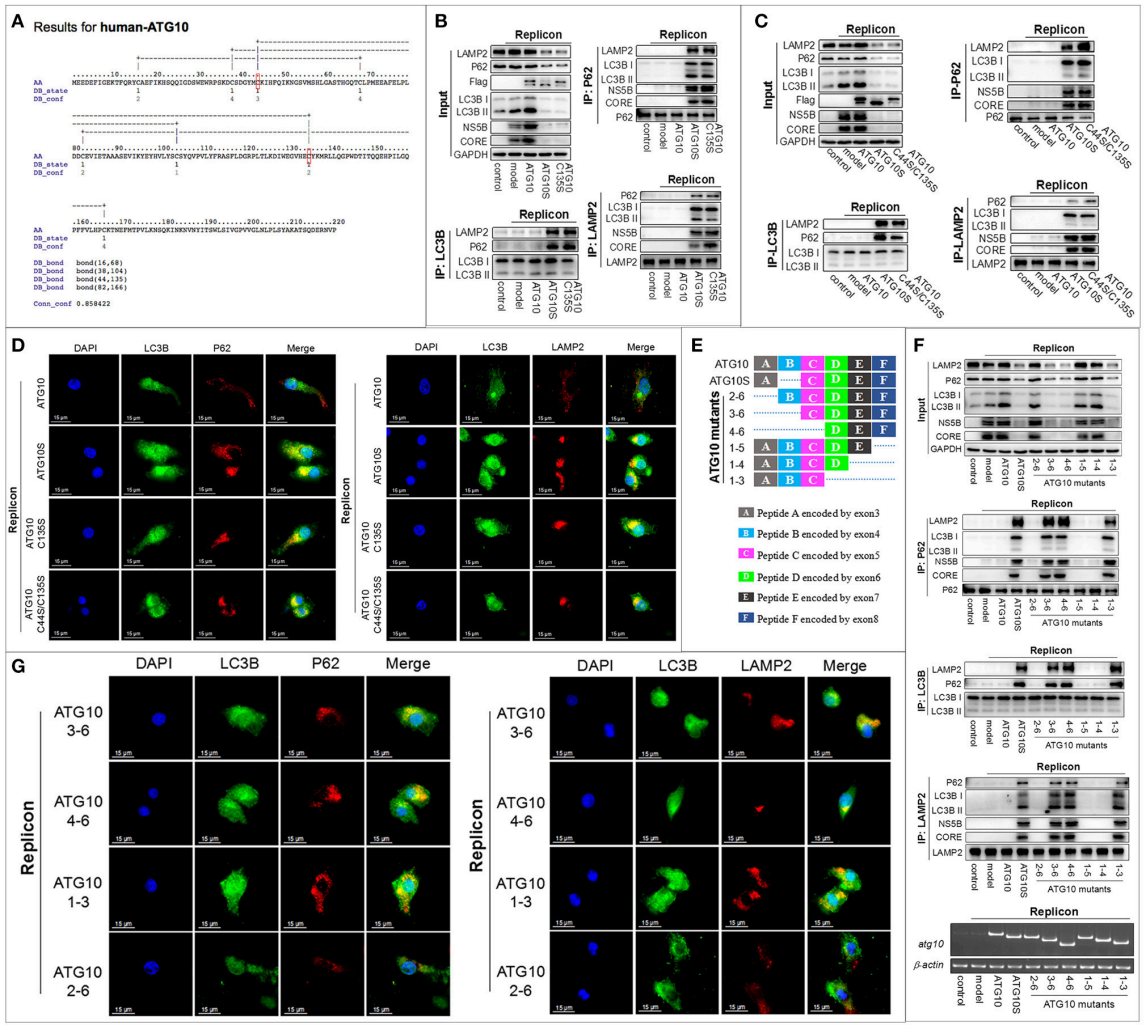
Cys^44^ and Cys^135^ are crucial for the transformation of the ATG10 protein structure and function. **(A)** The prediction of intrachain disulfide bonds in the ATG10 protein online (http://disulfind.dsi.unifi.it). Cys^44^ and Cys^135^ are indicated by a red box, respectively. **(B, C)** In the HCV-subgenomic replicon cells with ATG10, ATG10S, and ATG10^C135S^ overexpression, respectively **(B)**, or with ATG10, ATG10S, and ATG10^C44S/C135S^ overexpression, respectively **(C)**, co-immunoprecipitation with anti-LC3B or anti-P62 antibody shows the interactions among LC3B, P62, and LAMP2, and co-immunoprecipitation with anti-LAMP2 antibody shows the interaction between HCV subreplicon proteins (NS5B and CORE) with autophagolysosomes. Western blotting analysis shows the levels of autophagy flux proteins and HCV CORE and NS5B. **(D)** Subcellular co-localization of P62-LC3B and LC3B-LAMP2 among ATG10, ATG10S, ATG10^C135S^, and ATG10^C44S/C135S^ overexpression in the HCV subreplicon cells by immunofluorescence. **(E)** The diagram of a series of N- or C-terminally truncated ATG10 mutants based on six peptides of the ATG10 protein. The dotted lines represent deletion exons. **(F)** Co-immunoprecipitation with anti-LC3B, anti-P62, and anti-LAMP2 antibodies shows the interaction among autophagy proteins (P62, LC3B, and LAMP2) and the HCV subreplicon proteins (NS5B and CORE). Input result shows the effect on autophagy flux and HCV subreplicon duplication in HCV-subgenomic replicon cells plus *atg10, atg10s, atg10*^1−3^, *atg10*^1−4^, *atg10*^1−5^, *atg10*^2−6^, *atg10*^3−6^, or *atg10*^4−6^. **(G)** Immunofluorescence analysis shows the subcellular colocation of P62-LC3B and LC3B-LAMP2 in HCV-subgenomic replicon cells plus *atg10*^1−3^, *atg10*^2−6^, *atg10*^3−6^, or *atg10*^4−6^. Scale bars, 15 μm.

Further, since –SH of cysteine can participate in the disulfide bond to maintain the structure and functions of proteins, we asked whether the Cys^44^ forms an intramolecular disulfide bond with any of other cysteine residues in ATG10. We predicted the disulfide bonds of the ATG10 protein online (http://disulfind.dsi.unifi.it) which showed four tentative disulfide bonds within the ATG10 sequence (Figure 4A). Among the four, Cys^44^ was predicted to link Cys^135^ to form a disulfide bond. We therefore examined whether the tentative Cys^44^-Cys^135^ disulfide bond played a crucial role by constructing two ATG10 mutants in which Cys^135^ was changed into serine (ATG10^C135S^) and double substitution of Cys^44^ and Cys^135^ by serine (ATG10^C44S/C135S^). As shown in Figures 4B–D, ATG10^C135S^ and ATG10^C44S/C135S^ could trigger complete autophagy and suppress HCV duplication by promoting autophagy flux and lysosomal degradation of HCV proteins, similar to ATG10S. Next, we speculated whether the disruption of the tentative disulfide bond of Cys^44^-Cys^135^ produced new disulfide bonds in an intrachain or interchain, which could lead to ATG10S-like effects. However, given that ATG10^C44S/C135S^ overexpression presented the same effects as ATG10^C44S^ or ATG10^C135S^ on autophagy flux and on HCV replication (Figures 4B–D; Figure S4D), we inferred that the ATG10S-like effects did not result from the formation of a new intrachain or interchain disulfide bond by Cys^44^ or Cys^135^.

To further confirm the role of Cys^44^ and Cys^135^ and exclude the potential influence of other cysteines, we constructed a series of N- or C-terminally truncated ATG10 mutants, with six peptides deleted from N- or C-terminal sequentially (Figure 4E). In all, there are eight cysteines scattered in the peptide A–E of the native ATG10: Cys^16^ in peptide A, Cys^38^, Cys^44^ and Cys^68^ in peptide B, Cys^82^ and Cys^104^ in peptide C, Cys^135^ in peptide D, and Cys^166^ in peptide E. Through the same experiments, we clearly observed that the ATG10^3−6^, ATG10^4−6^, and ATG10^1−3^ mutants could induce complete autophagy and suppress HCV duplication, much like ATG10S (Figures 4F,G; Figure S4G), and the three ATG10 mutants were involved in Cys^44^ or Cys^135^ loss, suggesting the importance of Cys^44^ and Cys^135^. The other three truncated mutants (ATG10^2−6^, ATG10^1−4^, and ATG10^1−5^ lost Cys^16^, Cys^166^, and none, respectively) did not show the ATG10S-like effects, excluding Cys^16^ and Cys^166^ correlated to ATG10S-like function. With respect to the other four cysteines (Cys^38^, Cys^68^, Cys^82^, and Cys^104^), the overexpression of ATG10^3−6^ and ATG10^4−6^ in HCV-subreplicon cells showed the same roles as ATG10S though there is a peptide C difference between them, indicating that the presence or absence of Cys^82^ and Cys^104^ did not affect the action of the two ATG10 mutants. Thus, Cys^82^ and Cys^104^ were ruled out. Peptide B contained three cysteines, Cys^38^, Cys^44^ and Cys^68^, and based on the results shown in Figures 1B,C, Cys^38^ (lost in ATG10^Δ1^) and Cys^68^ (lost in ATG10^Δ6^) did not have similar functions as ATG10S. Therefore, we believe that loss of Cys^44^ and/or Cys^135^ was crucial for the conversion of the ATG10 protein structure and function. Furthermore, our results suggest that the other cysteines may not be involved in the ATG10S-like roles in autophagy flux and the suppression of HCV duplication.

### Roles of ATG10 with Cys^44^ and/or Cys^135^ mutation on HCV inhibition was confirmed by HCV virion infection

**Figure 5:**
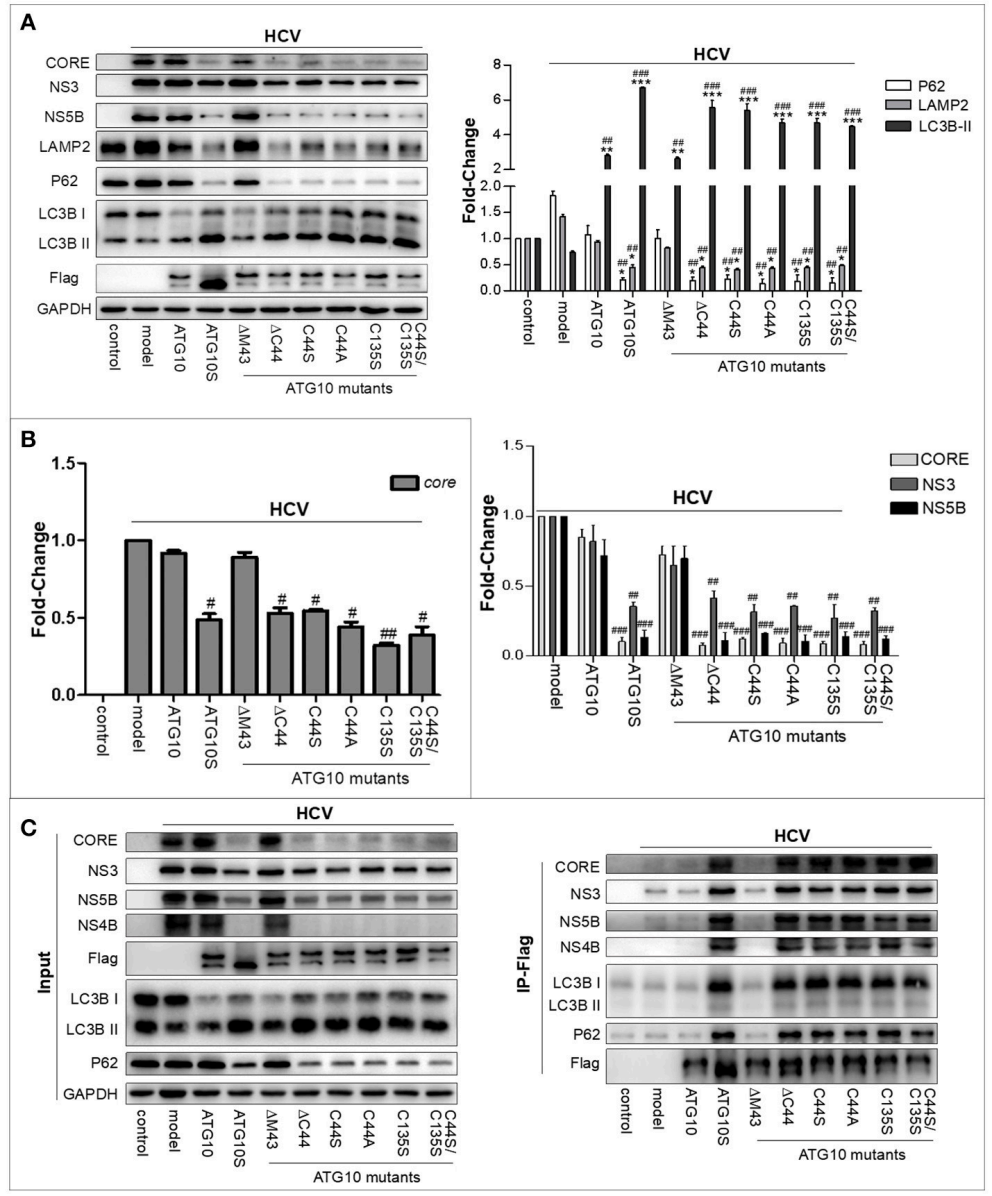
ATG10 without Cys^44^ and/or Cys^135^ downregulates HCV replication in HCV virion-infected cells. **(A)** The role of ATG10 without Cys^44^ and/or Cys^135^ on autophagy flux and HCV replication compared with ATG10^ΔM43^, ATG10, ATG10S, and the HCV model only, by Western blotting. **(B)** qRT-PCR results show that HCV core RNA was reduced by overexpression of ATG10 without Cys^44^ and/or Cys^135^ compared with ATG10^ΔM43^, ATG10, ATG10S, and the model only. *P < 0.05, **P < 0.01, ***P < 0.001 vs. Ctrl; ^#^P < 0.05, ^*##*^P < 0.01, ^*###*^P < 0.001 vs. the HCV model. All of the data are mean ± SD (*n* = 3). **(C)** Co-immunoprecipitation with anti-Flag antibody shows the interaction between HCV proteins (CORE, NS3, NS4B, and NS5B) and autophagy proteins (P62 and LC3B) with ATG10S and ATG10 mutants compared with the native ATG10 and ATG10^ΔM43^ in the HCV subreplicon cells.

To examine the effect of the Cys^44^ and Cys^135^ mutations in ATG10 on full HCV virion replication, Huh7.5 cells infected with HCV virion (HCV2a, J6/JFH/JC) were transfected with the ATG10 mutants ATG10^ΔC44^, ATG10^C44S^, ATG10^C44A^, ATG10^C135S^, and ATG10^C44S/C135S^ in advance, respectively, and compared with ATG10^ΔM43^, ATG10, ATG10S, and the HCV model only. The results showed that NS3, NS5B, and CORE proteins were decreased significantly in the ATG10 mutant overexpressions, except in ATG10^ΔM43^ and ATG10 groups as depicted by Western blotting (Figure 5A). In addition, HCV core RNA was notably reduced by the overexpression of these ATG10 mutants, except in ATG10^ΔM43^ and ATG10 groups using qRT-PCR (Figure 5B). These results indicated that these ATG10 mutants without Cys^44^ and/or Cys^135^ were efficient in suppressing the replication of HCV virion in the full-length virus infection model. Correspondingly, the levels of P62 and LAMP2 proteins were reduced, while LC3B-II was increased significantly (Figure 5A). These data suggested that ATG10 lacking Cys^44^ and/or Cys^135^ might promote complete autophagy. Further, Co-IP results verified the interaction among HCV proteins (structural protein CORE and non-structural proteins including NS3, NS4B and NS5B), autophagy flux proteins (LC3B and P62) and ATG10 mutants using anti-Flag antibody, just as the results in the HCV subreplicon model (Figure 5C). These results suggest that HCV virion products also can be digested by efficient autophagic machinery driven by ATG10S or by the mutants of ATG10 with Cys^44^ and/or Cys^135^ mutation, and demonstrate that the hydrosulfuryls (-SH) of Cys^44^ and Cys^135^ are crucial for ATG10, and that loss of the hydrosulfuryls on Cys^44^ and Cys^135^ can suppress HCV genomic replication in the conventional HCV virion-infected cells.

### Anti-HCV role of ATG10S and ATG10 with Cys^44^ and/or Cys^135^ mutation is IL28A-dependent

**Figure 6:**
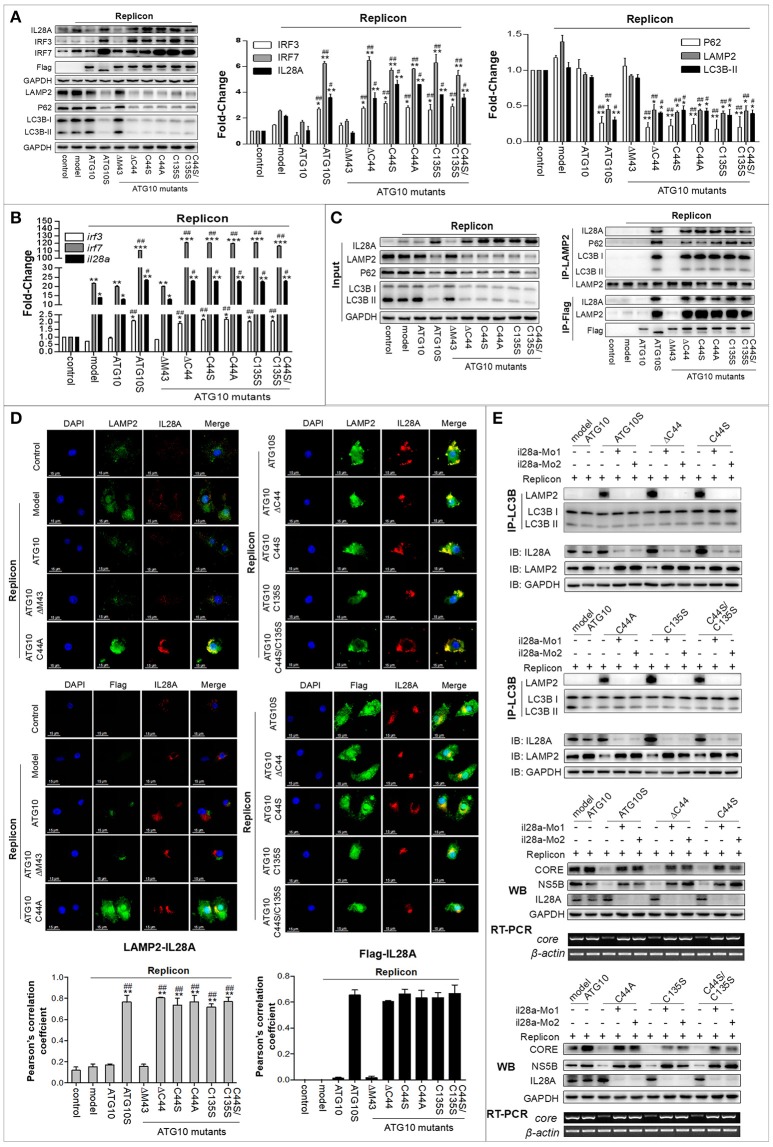
IL28A expression was activated by ATG10S and ATG10 mutants with Cys^44^-Cys^135^ mutation, and mediated autolysosome formation and HCV degradation. **(A)** Protein levels of the immune factors (IL28A, IRF3, and IRF7) and autophagy flux-related proteins (LC3B, P62, and LAMP2) were analyzed by Western blotting. **(B)** Transcription levels of IL28A, IRF3, and IRF7 were tested by qPCR. **(C)** Co-Immunoprecipitation showed the interactions among LAMP2, IL28A, P62, and LC3B using anti-LAMP2 antibody, and the interactions among IL28A with ATG10 mutant proteins and LAMP2 using anti-Flag antibody (labeling the ATG10, ATG10S, and the ATG10 mutants). **(D)** Immunofluorescence analysis shows co-localization of IL28A with LAMP2 by using anti-IL28A and anti-LAMP2 antibodies and co-localization of ATG10 mutants with IL28A by using anti-Flag and anti-IL28A antibodies in the HCV subreplicon cells. **(E)** The interactions between autophagosomes and lysosomes were disappeared in IL28A-knockdown cells by immunoprecipitation with anti-LC3B antibody (upper two panels). The replication of HCV subgenomic replicon was restored in IL28A-knockdown cells detected by Western blotting and RT-PCR tests in which HCV CORE and NS5B proteins and *core* RNA were obviously elevated via IL28A downregulation (lower two panels). Scale bars, 15 μm. *P < 0.05, **P < 0.01, ***P < 0.001 vs. Ctrl; ^#^*P* < 0.05, ^*##*^*P* < 0.01, ^*###*^*P* < 0.001 vs. the replicon model.

In our previous study, ATG10S was inferred to suppress HCV RNA amplification via two pathways: activing the innate immune system, and restoring autophagy flux by driving autophagosomes to combine with lysosomes with the help of IL28A (31). The above results suggested that ATG10 mutants without Cys^44^ and Cys^135^ suppress HCV replication by promoting complete autophagy. However, whether these ATG10 variants can also activate an innate immune response similar to ATG10S, and whether the IL28A protein participates in the fusion of lysosomes to autophagosomes in these groups of ATG10 mutants' overexpression are unclear. Therefore, we assessed the expression of some innate immunity-related genes, such as type 3 interferon (*il28a*) and interferon regulatory factors (*irf3* and *irf7*) by Western blotting and qPCR analysis. As shown in Figures 6A,B, the levels of IL28A/*il28a*, IRF3/*irf3*, and IRF7/*irf7* were notably up-regulated in the groups transfected with ATG10S or ATG10 mutants ATG10^ΔC44^, ATG10^C44S^, ATG10^C44A^, ATG10^C135S^ and ATG10^C44S/C135S^, respectively, compared with ATG10, the model, and ATG10^ΔM43^ mutant in the HCV subgenomic replicon cells. To learn whether the up-regulation of the three immune factors is related to autophagy or to the mutants' non-autophagy function, the cells were treated by rapamycin, an autophagy activator, and Western blotting test was used to detect these proteins. The results showed that rapamycin did not change levels of IL28A, IRF3, and IRF7; and once again, these proteins were raised in ATG10S and the groups of ATG10^ΔC44^, ATG10^C44S^, ATG10^C44A^, ATG10^C135S^, and ATG10^C44S/C135S^ with and without rapamycin treatment, indicating that the up-regulation of the three proteins was not related to autophagic activation, but promoted by ATG10S and the ATG10 mutants without Cys44 and/or Cys135 (Figure S6A). Co-IP results verified the interaction of lysosomes with IL28A and the autophagy flux proteins (LC3B and P62) using anti-LAMP2 antibody, and the interaction of the ATG10 mutants with LAMP2 and IL28A using anti-Flag antibody (Flag was fused to N-terminal of ATG10 mutants) in the HCV subgenomic replicon cells co-transfected with the ATG10 mutants (Figure 6C). The results offered positive evidence that IL28A combined with LAMP2 and with the ATG10 variants without Cys^44^ or/and Cys^135^. The results of cell immunofluorescence double staining by using anti-Flag, anti-LAMP2, and anti-IL28A antibodies showed that IL28A indeed co-localized with LAMP2 and the ATG10 mutants in a high Pearson's coefficient (Figure 6D). Conversely, endogenous il28a-knockdown by morpholino oligos (il28a-MO1 and il28a-MO2) inhibited the interaction of LC3B with LAMP2 and rescued the replication of HCV sugenomic replicon, in which HCV *core* RNA, CORE and NS5B proteins were significantly upregulated (Figure 6E). These suggest that autophagy flux was blocked and the fusion of autophagosomes to lysosomes was inhibited without IL28A existence, even though ATG10S and the ATG10 mutants without Cys^44^ or/and Cys^135^ were still overexpressed. Therefore, the IL28A protein may play a significant linker role between autophagosomes and lysosomes; and an anti-HCV role of ATG10S and the ATG10 mutants without Cys^44^ or/and Cys^135^ may be IL28A-dependent and partly resulted from their non-autophagy function and partly from their participation in autolysosome formation.

### ATG10S and ATG10 mutants without Cys^44^ or/and Cys^135^ may function as transcription factors for innate immunity response genes

**Figure 7:**
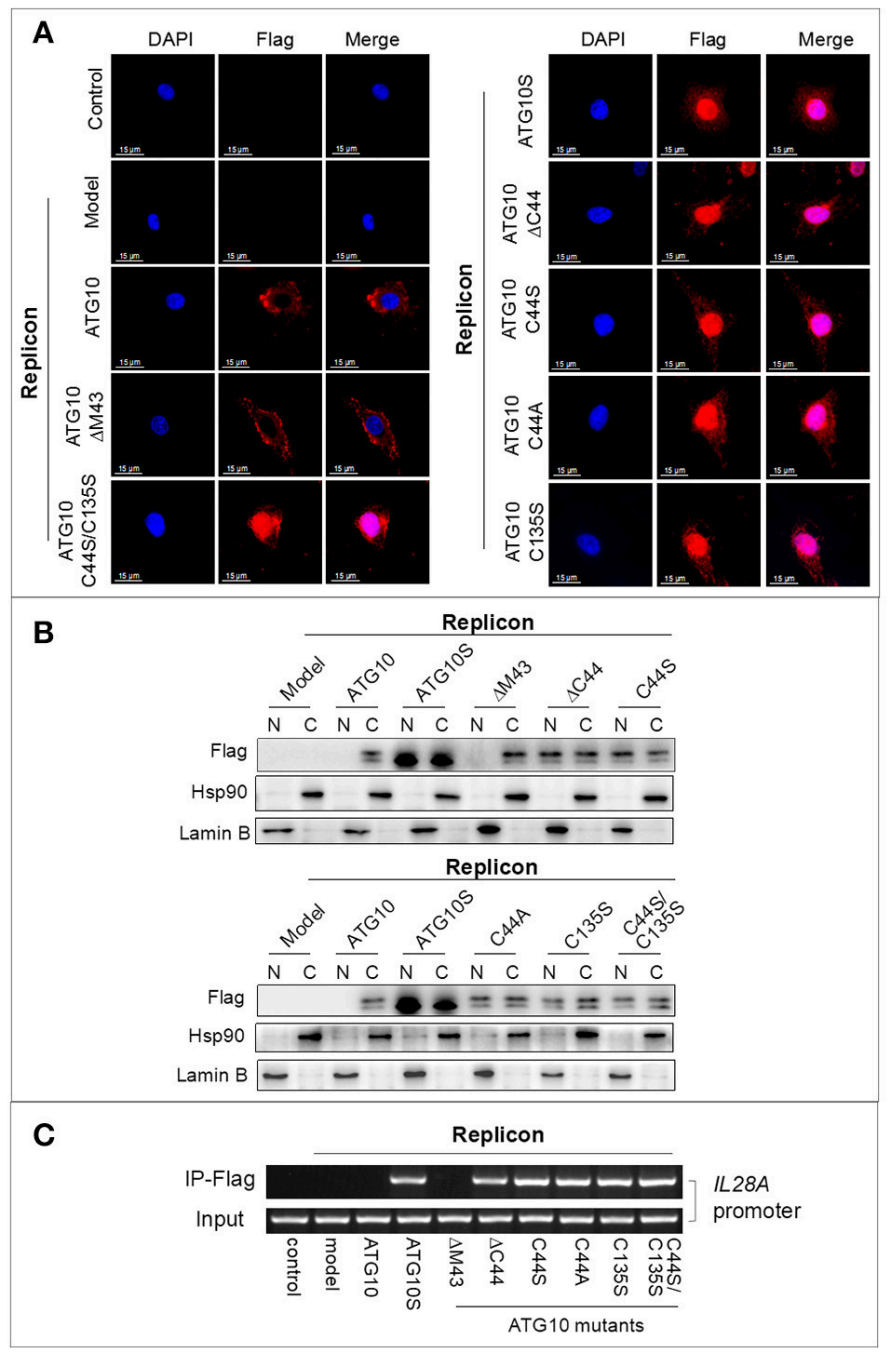
ATG10 mutants with Cys^44^ and/or Cys^135^ mutation function as a kind of transcription factors. **(A,B)** Immunofluorescence by using anti-Flag antibody and nuclear–cytoplasmic fractionation analysis show the nuclear translocation of the ATG10 mutants without Cys^44^ or/and Cys^135^. Scale bars: 15 μm. **(C)** The ATG10 mutants and ATG10S docking to *IL28A* promoter were confirmed by ChIP assay with anti-Flag, in which.a 400 bp fragment of IL28A promoter was amplified from the precipitates of the groups of ATGS and the ATG10 mutants without Cys^44^ or/and Cys^135^ compared with four groups of the control, the subreplicon model, and the model plus native ATG10 or plus ATG10^ΔM43^.

ATG10S and ATG10 mutants with Cys^44^ and/or Cys^135^ mutation promoted autophagy flux and suppression of HCV duplication, which was functionally different from the native ATG10, and they significantly up-regulated IL28A, IRF3, and IRF7 in both transcription and protein levels (Figures 6A,B). This potentially underscores the important roles of ATG10S and the ATG10 mutants in immune modulation in antiviral defense as key transcription regulating factors of interferons (IFNs) and IFN-stimulated genes (ISGs). As shown in Figures 7A,B, the results of cell immunofluorescence and nuclear–cytoplasmic fractionation showed that ATG10S and the ATG10 mutants could not only stay in cytoplasm but also dock into the cell nucleus, which was dependent on Cys^44^ and/or Cys^135^ mutation or absence in the ATG10 protein. Further, we examined their DNA binding activity at *il28a* promoter using a ChIP assay; the precipitates pulled down by Flag-antibody were detected to contain *il28a* promoter fragment in the groups of ATG10S and ATG10 mutants without Cys^44^ and/or Cys^135^, but no *il28a* promoter fragment in the precipitates from both ATG10 and ATG10^ΔM43^ groups (Figure 7C). These data suggest that the ATG10S and the ATG10 mutants probably play the roles of transcriptional factors to initiate the expression of anti-HCV immunological genes. However, the exact mechanism needs to be further studied.

### Transformation of ATG10 function is dependent of its steric conformation change

**Figure 8:**
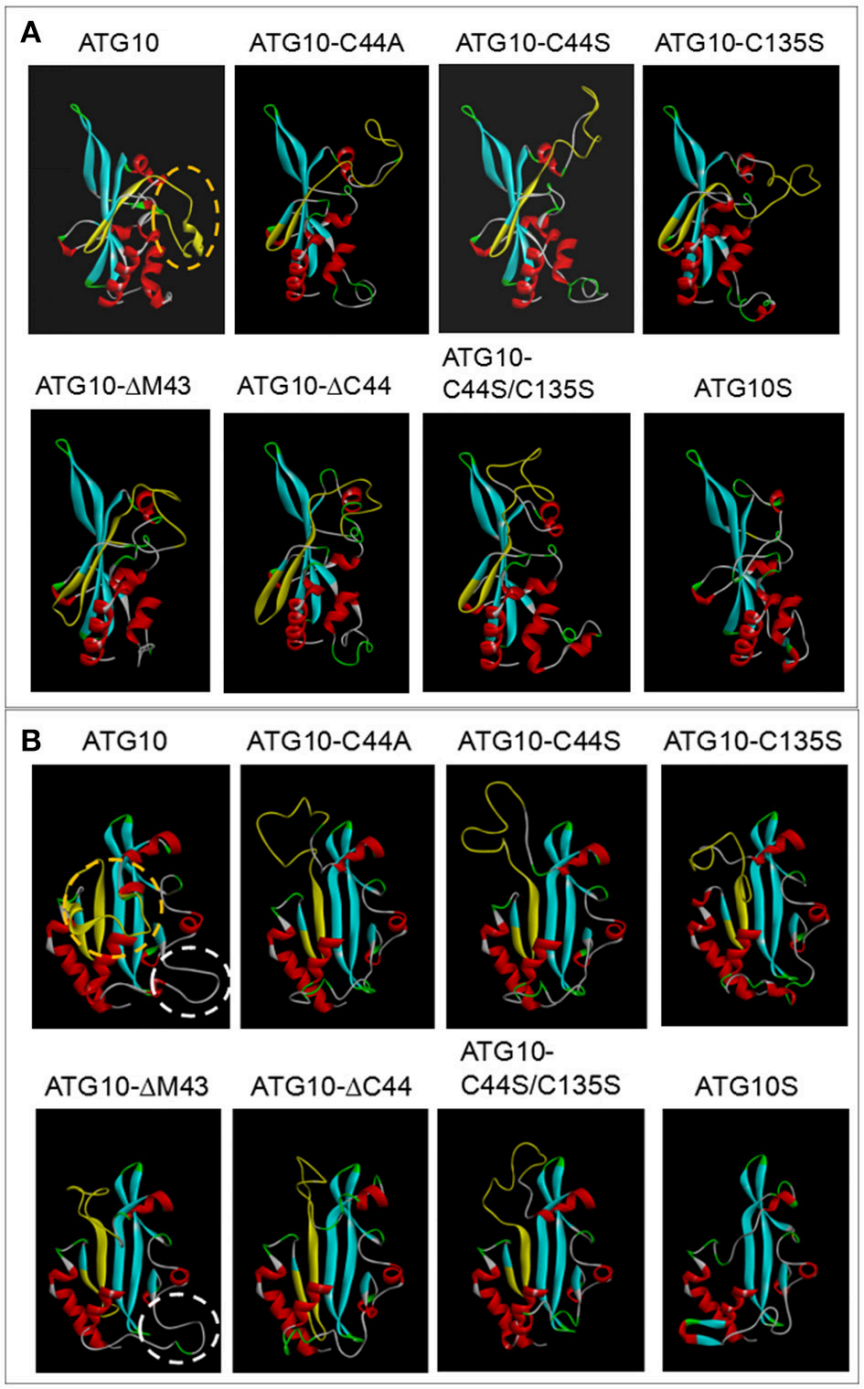
Comparison analysis of three dimensional structures between ATG10 and its mutants. **(A,B)** Homology modeling of ATG10 protein and its mutants were performed using a template 4gsk.1.c online (http://swissmodel.expasy.org/). Peptide B of ATG10 was indicated by yellow structure. In the lateral view **(A)** and the front view **(B)** of the ATG10 protein, the orange dashed rings show the random ring of the peptide B acting as a curtain sealing the beta-sheet in the front side of ATG10 protein. The white dashed rings indicate an extension loop in both ATG10 and ATG10^ΔM43^ proteins. The other five ATG10 mutants and ATG10S proteins do not have the “curtain” and the ring.

To further explore whether the disulfide bond of Cys^44^-Cys^135^ actually exists and, if so, whether it controls ATG10 function transformation, we modeled the steric conformations of ATG10 and its mutant proteins using the SWISS MODEL software online (http://swissmodel.expasy.org/) and the template 4gsk.1.c (hetero-2-2-mer, and by a method of X-RAY DIFFRACTION 2.90 Å) (37–39), and performed comparative analysis of the ATG10 steric structures. The results showed some features among the proteins: first, they may be involved in an Atg7-Atg10 crosslinked complex; second, no intrachain disulfide bond was found in these ATG10 proteins and the distance between Cys^44^ and Cys^135^ appeared too far to connect, which is different from the previous disulfide-bond prediction (http://disulfind.dsi.unifi.it); and third, peptide B as a curtain shields the front β-sheet panel of ATG10, but the front β-sheet panel is open in ATG10S and the ATG10 mutants except ATG10^ΔM43^ (Figure 8A). Another discrepancy is that an extension loop (at Lys^167^-Leu^176^) appeared in ATG10 and ATG10^ΔM43^ proteins but not in the other mutants and ATG10S (Figure 8B), which coincidence with their differential roles on HCV replication and on autophagy flux. These results indicated that both the peptide B shelter position and the extended loop may be involved in blocking autophagy flux and lysosomal degradation of HCV genome; mutation or loss of Cys^44^ or/and Cys^135^ made ATG10 steric conformation change toward a ATG10S-like structure, leading to the similar ATG10S functions.

## Discussion

The current data demonstrate the importance of two sulfhydryl groups on Cys^44^ and Cys^135^ in ATG10 protein, and reveal a mechanism underlying the differential roles on HCV replication and autophagy flux between ATG10 and ATG10S. Previous studies reported that ATG10 as an E2-like ubiquitination enzyme combined with ATG7 (35, 40, 41) and ATG5-ATG12 (4), as well as with immunity-associated GTPase family M (IRGM) that is known to be involved in virus and bacterial infections and interferon regulation (36, 42). This suggests that the native ATG10 protein generally exists as an oligomer manner in the autophagy process. Recently, our studies showed that the two isoforms of ATG10 and ATG10S had distinct roles on HCV replication and autophagy flux. The two ATG10 isoforms have a 36-amino acids difference encoded by exon-4 of atg10 transcript variant 3 sequence in human chromosome 5 (31). In the present study, we aimed to understand what caused the differential effects between ATG10 and ATG10S. Focusing on peptide B, which is absent in ATG10S, and using sequential deletion per six amino acids in each unit, and sequential amino acid deletion in unit-2, we found that the Cys^44^ of the ATG10 protein is a key amino acid. Furthermore, we investigated whether the differential roles were dependent on –SH group of Cys^44^. And, substitution experiments of the cysteine by serine (the only difference is –OH replacing -SH) or by alanine (the only difference is –H replacing -SH) confirmed that the ATG10 mutants played similar roles as ATG10S. Therefore, we inferred that the function characteristics of the ATG10 variants and ATG10S in HCV-inhibition and promoting autophagy flux were only associated with Cys^44^ sulfhydryl deletion. Next, we posited that a disulfide bond might mediate the opposing actions. A disulfide bond between Cys^44^ and Cys^135^ was predicted online (http://disulfind.dsi.unifi.it); and sulfhydryl deletion of Cys^44^ or/and Cys^135^ promoted the formation of autophagolysosomes and HCV subgenomic replicon degradation, accompanied by the interaction of NS5B and CORE with P62 and with LAMP2 (lysosome) in the ATG10 mutant-overexpressing cells. These results demonstrated that HCV products were delivered to autolysosomes and were degraded. Further, these findings were confirmed in the HCV virion-infection cells. Co-IP results indicated that these HCV proteins were carried into autophagosomes for digestion, too; particularly, HCV NS4B was decreased more significantly than CORE, NS3 and NS5B (Figure 5C). In consideration of NS4B being involved in intracellular membrane rearrangement and anchoring HCV replication complex to the membrane web (43–45) for facilitating HCV replication, the NS4B level decrease means that the membrane web was disrupted, which might aggravate degradation of HCV replication machinery under overexpression of the ATG10 mutants without Cys^44^ and/or Cys^135^.

To further identify whether the disulfide bond of Cys^44^-Cys^135^ actually exists, we investigated protein conformations of ATG10 and its mutants by homology modeling and comparative analysis. The results rejected existence of a disulfide bond between Cys^44^ and Cys^135^ because the two cysteines were apart too far to connect, and no any intrachain disulfide bond was predicted in these ATG10 proteins. This suggests that ATG10 function change might not be involved in intrachain disulfide bond in ATG10. Moreover, the peptide B “curtain” structure and the extended loop in ATG10 and ATG10^ΔM43^ are absent in ATG10S and the ATG10 mutants lacking Cys^44^ and/or Cys^135^. Thus, we speculate that mutation or loss of Cys^44^ or/and Cys^135^ made ATG10 steric conformational change toward a ATG10S-like structure, rather than old disulfide bonds were disrupted and new ones reformed, leading to the similar ATG10S functions.

Another significant finding is the activation of the innate immunity response by the overexpression of the ATG10 variants in the HCV subgenomic replicon cells. In our previous studies, ATG10S activated the innate immune response in HCV subgenomic replicon cells via up-regulating five innate immunologic factors (i.e., IL28a, IRF-3, IRF-7, TLR3, and TLR7). In this study, it was verified that the ATG10 mutants without Cys^44^ and Cys^135^ also elevated the levels of *il28a*/IL28a, *ifr-3*/IRF-3, and *irf-7*/IRF-7. IRF-3 and IRF-7 could induce IFNs expression as interferon regulatory factors (46, 47) by nuclear-translocation upon virus infection (48). In this study, the nuclear translocation and nuclear–cytoplasmic fractionation assays indicated that the ATG10 mutant proteins surprisingly translocated into the nucleus much like the ATG10S protein, but ATG10 and ATG10^ΔM43^ did not. Moreover, We found ATG10S and the ATG10 mutants without Cys^44^ and/or Cys^135^ could combine with *IL28A* promoter by ChIP assay. These results raised the possibility that ATG10 might act as a latent transcription factor after losing its -SH group on Cys^44^ and/or Cys^135^. This may be due to the varied structure of the ATG10 mutants, which may increase their affinity to some particular nuclear transport receptors (NTRs), resulting in triggering nuclear migration of the ATG10 variants. The molecular mechanism of nuclear translocation of the ATG10 variants needs to be further investigated.

IL28A belongs to the IFN-λ family and it is categorized as type III IFN, which is a group of potent antiviral cytokines (49) that play a critical role in the innate defense system. Previous studies have shown that IL28A inhibits HCV replication *in vitro* (50, 51) via inducing the transcription of IFN-stimulated genes (ISGs) to control viral infection and replication (52). Our previous studies demonstrated that IL28A might mediate the autophagosome docking to lysosomes, and ATG10S promoted the formation of autophagolysosomes with the help of IL28A. In this study, using two il28a-MOs that inhibited the expression of endogenous il28a, we confirmed the crucial role of IL28A for the ATG10 variants lacking -SH group on Cys^44^ and/or on Cys^135^. These ATG10 mutants could drive the fusion of autophagosomes with lysosomes under the existence of IL28A; however, the replication of HCV sugenomic replicon was restored without the help of IL28A, even though the ATG10 mutants without Cys^44^ and/or Cys^135^ overexpression. These results suggesting that the ATG10 variants with IL28A can mimic ATG10S to inhibit HCV subgenome replication via driving autophagy flux and autophagolysosome degradation. We speculate that the loss of the –SH group in Cys^44^ and Cys^135^ may cause ATG10-related hetero-oligomer dissolution and changes, which increased the likelihood of ATG10 recognizing and interacting with IL28A, and of its nuclear translocation based on the experimental evidence in Figure 7. These changed functions of ATG10 proteins are similar to the ATG10S function. On the other hand, the two -SH groups are probably necessary for the native ATG10 structure and functions.

**Figure 9:**
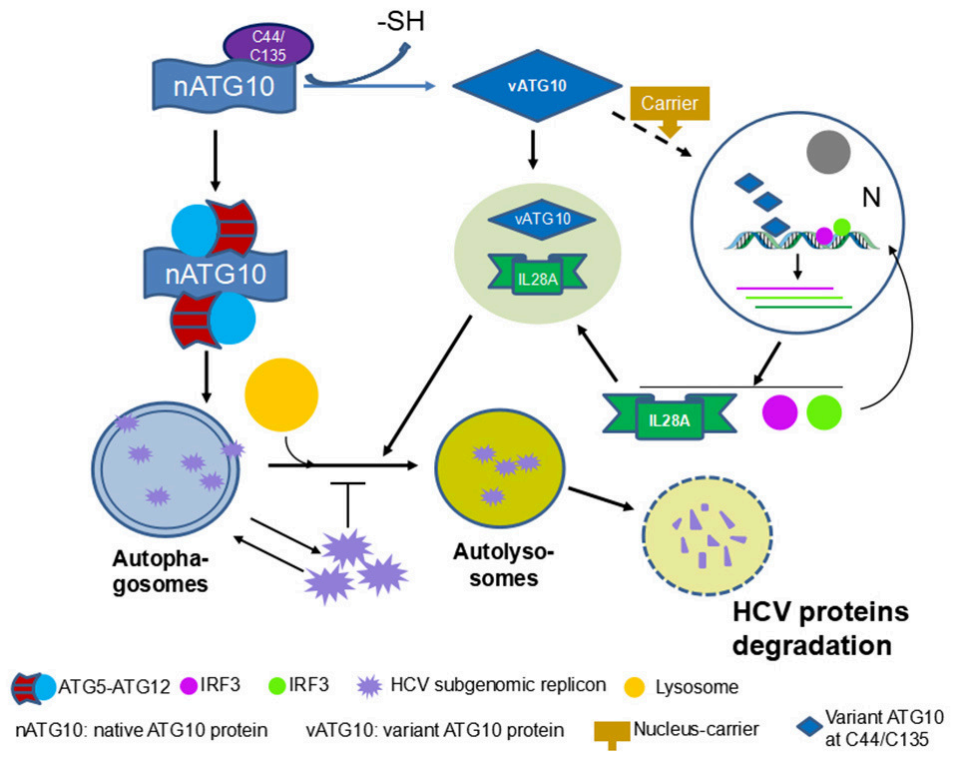
Overview of ATG10 function change by loss of -SH group on Cys^44^ and/or Cys^135^.

In summary, our findings suggest that the absence of peptide B in ATG10S is the causative factor for ATG10S anti-HCV and the promotion of autophagy flux. As summarized in Figure 9, Cys^44^ is a key amino acid of peptide B and loss of –SH group on Cys^44^ and/or Cys^135^ may significantly cause ATG10 conformational change, which results in ATG10S-like functions.

## Author contributions

J-PZ conceived and designed the project. M-QZ performed the most of the experiments and treated data. M-QZ and J-PZ analyzed the results and data, and wrote the manuscript. Z-GP provided the HCV virion, Huh7.5 cell strain and the method for HCV infection. J-RL performed the experiments of the HCV virion infection.

### Conflict of interest statement

The authors declare that the research was conducted in the absence of any commercial or financial relationships that could be construed as a potential conflict of interest.

## Abbreviations

ATG10: autophagy related protein 10
HCV: hepatitis C virus
IL28A: interleukin-28A
IRF-3: interferon regulatory factor 3
IRF-7: interferon regulatory factor 7
LC3B: microtubule-associated protein 1 light chain 3 beta
LAMP2: lysosomal associated membrane protein 2
P62: sequestosome 1
Co-IP: Co-immunoprecipitation
IF: immunofluorescence.
