# Supplementary material for: Differential Effects of Autophagy-Related 10 Protein on HCV Replication and Autophagy Flux Are Mediated by Its Cysteine44 and Cysteine135
Source: Front Immunol. 2018 Sep 24;9:2176. doi: 10.3389/fimmu.2018.02176 (PMC6165859; doi:10.3389/fimmu.2018.02176)
Supplement: Supplementary file 1 [file Data_Sheet_1.PDF]

Supplementary Material (387041)

Differential effects of Autophagy-related 10 protein on HCV replication and autophagy flux are mediated by its Cysteine[44] and Cysteine[135]

Miao-Qing Zhang, Jian-Rui Li, Zong-Gen Peng, Jing-Pu Zhang*

*** Correspondence: Jing-Pu Zhang**: zhangjingpu@imb.pumc.edu.cn or zjp5577@126.com

Supplementary Figures

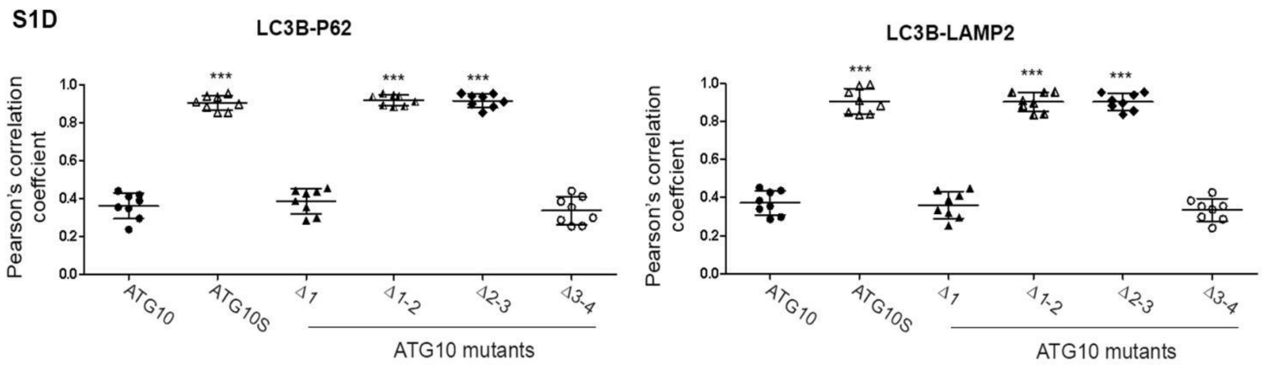

Figure S1D. The scatter plot of Pearson coefficients correspond to Figure 1D. Pearson coefficients of the LC3B-P62 conjugation and LC3B-LAMP2 conjugation were significantly elevated by ATG10S, ATG10$^{\Delta 1\text{-}2}$ or ATG10$^{\Delta 2\text{-}3}$ overexpression compared with ATG10, ATG10$^{\Delta 1}$, or ATG10$^{\Delta 3\text{-}4}$. ***P< 0.001 vs ATG10.

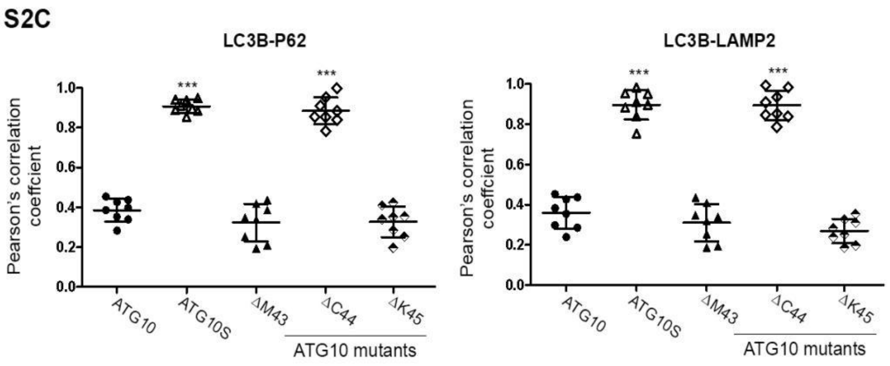

Figure S2C. The scatter plot of Pearson coefficients correspond to Figure 2C. LC3B-P62 and LC3B-LAMP2 conjugation was significantly enhanced by ATG10S or ATG10$^{\Delta C44}$ overexpression compared with ATG10, ATG10$^{\Delta M43}$, or ATG10$^{\Delta K45}$. ***P< 0.001 vs ATG10.

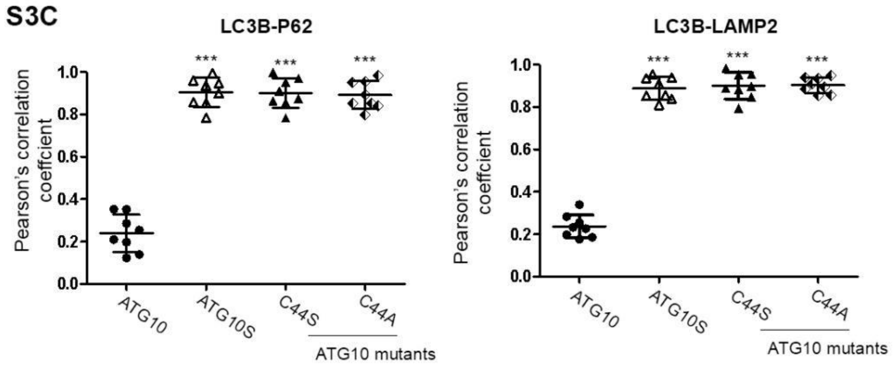

Figure S3C. The scatter plot of Pearson coefficients correspond to Figure 3C. Pearson coefficient of LC3B-P62 and LC3B-LAMP2 combination increased in ATG10S, $ATG10^{C44S}$ or $ATG10^{C44A}$ overexpression but not ATG10 in HCV replicon-cells. ***$P< 0.001$ vs ATG10.

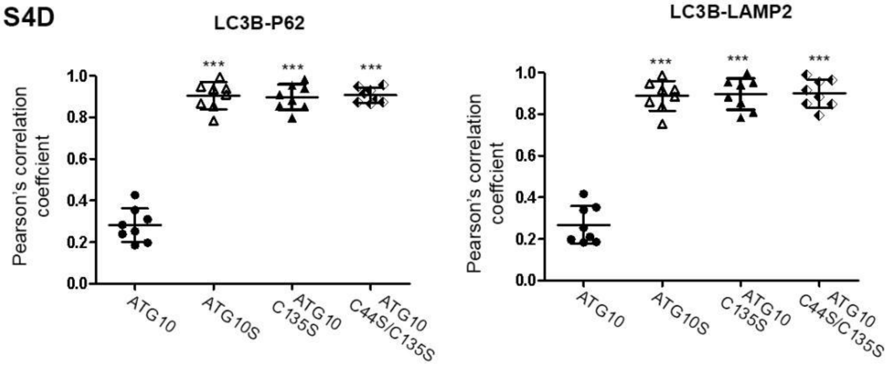

Figure S4D. The scatter plot of Pearson coefficients correspond to Figure 4D. LC3B-P62 and LC3B-LAMP2 conjugation was significantly enhanced by ATG10S, $ATG10^{C135S}$ or $ATG10^{C44S/C135S}$ overexpression but not ATG10. ***$P< 0.001$ vs ATG10.

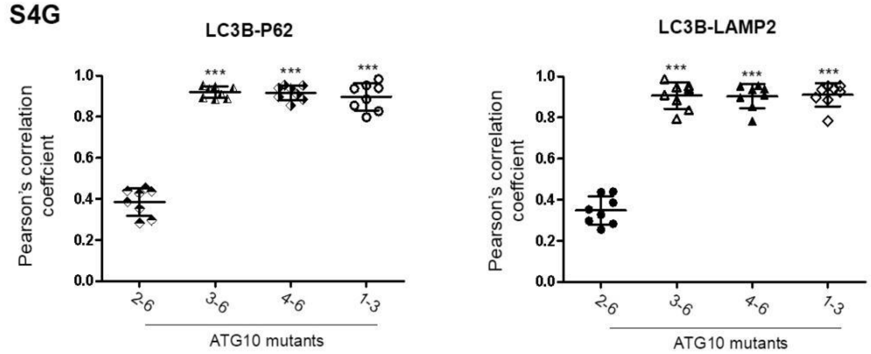

Figure S4G. The scatter plot of Pearson coefficients correspond to Figure 4G. $ATG10^{3-6}$, $ATG10^{4-6}$, or $ATG10^{1-3}$ elevated significantly the Pearson coefficient of the LC3B-P62 and LC3B-LAMP2 conjugation compare with $ATG10^{2-6}$ in the HCV replicon-cells. ***$P< 0.001$ vs $ATG10^{2-6}$.

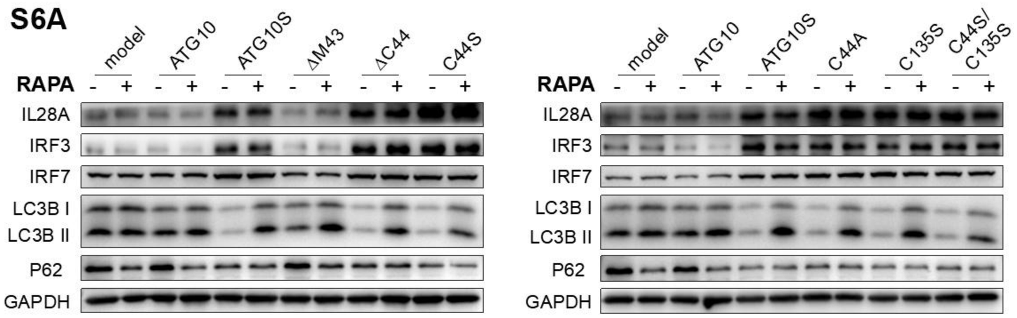

Figure S6A. Infuence of rapamycin on protein levels of IL28A, IRF3 and IRF7. RAPA treatment (50 nM) did not affect protein levels of IL28A, IRF3, and IRF7 in all the groups, but differentially increased LC3B-II level, particularly in the groups of ATG10S and the ATG10 mutants with cys absence at 44 and/or 135 site, and declined P62 level in the model, ATG10 and ATG10$^{\Delta M43}$ groups, and not changed in the other groups in which P62 level were at low level compared to the model without RAPA.
